# Structural and Functional Insight Into the Glycosylation Impact Upon the HGF/c-Met Signaling Pathway

**DOI:** 10.3389/fcell.2020.00490

**Published:** 2020-06-18

**Authors:** Xinyue Hu, Feiyu Tang, Peilin Liu, Taowei Zhong, Fengyan Yuan, Quanyuan He, Mark von Itzstein, Hao Li, Liang Weng, Xing Yu

**Affiliations:** ^1^College of Medicine, Hunan Normal University, Changsha, China; ^2^Center for Molecular Medicine, Xiangya Hospital, Central South University, Changsha, China; ^3^Key Laboratory of Model Animals and Stem Cell Biology in Hunan Province, Hunan Normal University, Changsha, China; ^4^Institute for Glycomics, Griffith University, Gold Coast, QLD, Australia; ^5^Biliary Tract Surgery Laboratory, Department of Hepatobiliary Surgery, Hunan Provincial People’s Hospital, The First Affiliated Hospital of Hunan Normal University, Changsha, China; ^6^Hunan Research Center of Biliary Disease, The First Affiliated Hospital of Hunan Normal University, Changsha, China; ^7^Key Laboratory of Molecular Radiation Oncology in Hunan Province, Central South University, Changsha, China

**Keywords:** HGF, c-Met, glycosylation, cancer, application

## Abstract

Upon interactions with its specific ligand hepatocyte growth factor (HGF), the c-Met signal is relayed to series of downstream pathways, exerting essential biological roles. Dysregulation of the HGF-c-Met signaling pathway has been implicated in the onset, progression and metastasis of various cancers, making the HGF-c-Met axis a promising therapeutic target. Both c-Met and HGF undergo glycosylation, which appears to be biologically relevant to their function and structural integrity. Different types of glycoconjugates in the local cellular environment can also regulate HGF/c-Met signaling by distinct mechanisms. However, detailed knowledge pertaining to the glycosylation machinery of the HGF-c-Met axis as well as its potential applications in oncology research is yet to be established. This mini review highlights the significance of the HGF-c-Met signaling pathway in physiological and pathological context, and discusses the molecular mechanisms by which affect the glycosylation of the HGF-c-Met axis. Owing to the crucial role played by glycosylation in the regulation of HGF/c-Met activity, better understanding of this less exploited field may contribute to the development of novel therapeutics targeting glycoepitopes.

## Introduction

Since the discovery of c-Met and its high-affinity cognate ligand HGF in 1980s (Nakamura and Mizuno, 2010), decades of research have disclosed that c-Met and HGF play significant roles in embryonic development, tissue regeneration and cell motility (Sakai et al., 2015; De Silva et al., 2017). Aberrant HGF/c-Met signaling has been well known to play central roles in tumorigenesis and cancer progression with poor prognosis, suggesting that the c-Met/HGF axis may be considered as a promising therapeutic target (Xiang et al., 2017; Cheng and Guo, 2019).

Glycosylation is a common type of protein post-translational modification, playing a critical role in determining protein function and undergoing profound changes in cancer (Fuster and Esko, 2005; Ferreira et al., 2018; Peixoto et al., 2019). It is noted that both HGF and c-Met are heavily glycosylated in their extracellular domains. Though biological activities of non-glycosylated HGF were not appreciably changed compared with glycosylated native HGF, at least in vitro assays, glycan contents of c-Met significantly influence its biological function (Fukuta et al., 2005; Chen et al., 2013). Glycoconjugates are defined as biological molecules comprised of a sugar portion linked to the aglycone part (typically proteins or lipids) (Pinho and Reis, 2015). Two major classes of glycoconjugates, heparan sulfate proteoglycans (HSPGs) and gangliosides, have been shown to regulate HGF/c-Met signaling. HSPGs, a group of glycoprotein ubiquitously found on the cell surface and in the extracellular matrix (ECM), seems to directly interact with HGF and act as a co-receptor to facilitate HGF/c-Met signaling (Zhou et al., 1998; Iscan et al., 2017). Meanwhile, regulation of c-Met activity by gangliosides is mediated by different mechanisms, depending on the composition of the ganglioside glycan chains (Oblinger et al., 2003; Kaucic et al., 2006). Interestingly, both HSPGs and some gangliosides have been reported to induce c-Met activation in the absence of HGF (Kaucic et al., 2006; Iscan et al., 2017). This mini review focuses on how glycosylation can influence the HGF-c-Met signaling pathway. In addition, we examine the potential of developing novel therapeutics targeting the HGF-c-Met axis by discussing the molecular mechanisms underlying the glycosylation modification.

## Structural and Functional Characteristics of HGF and c-Met

**FIGURE 1:**
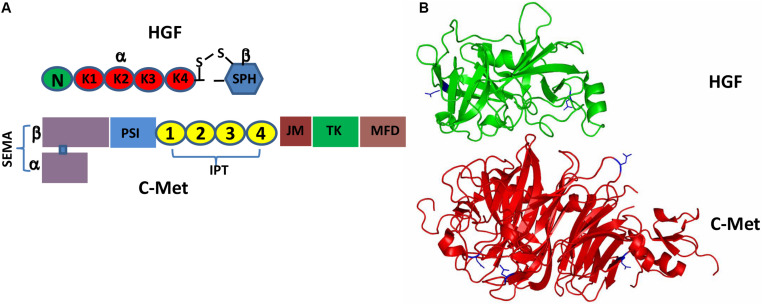
Schematic representation of the HGF/c-Met structures. **(A)** Pro-HGF is cleaved upon proteolytic activation between the SPH domain and the K4 domain to generate a mature HGF with disulfide-linked α-chain (the N-terminal and K1-4 domains) and β-chain (the SPH domain). Similarly, the heterogenous c-Met contains α-chain and β-chain forming a heterodimer; **(B)** 3D schematic ribbon representation of the HGF β-chain-c-Met SEMA domain complex structure (HGF in green and c-Met in red). *N*-linked glycosylation occurs at Asn566 and Asn653 in HGF as well as Asn45, Asn106, Asn149 and Asn202 in c-Met.

It has been more than three decades since HGF was originally discovered as a hormone-like substance in the serum that was highly mitogenic for hepatocytes (Nakamura et al., 1984; Oliveira et al., 2018). HGF is initially produced and secreted by stromal cells (mainly fibroblasts and smooth muscle cells) in the form of pro-HGF that is biologically inactive (Miyazawa et al., 1994, 1996; Jangphattananont et al., 2019). The single chain Pro-HGF is then proteolytically cleaved at Arg^494^ and Val^495^, generating mature HGF in a heterogeneous two-chain form (Miyazawa et al., 1993; Naldini et al., 1995; Kawaguchi and Kataoka, 2014). Biologically active HGF, a disulfide-linked heterodimer, comprises a heavy α-chain of four kringle domains (K1-K4) and an *N*-terminal hairpin loop and a light β-chain of the *C*-terminal serine proteinase homology (SPH) domain (Figure 1A; Donate et al., 1994; Organ and Tsao, 2011). Active c-Met is a disulfide-linked heterodimer consisting of an extracellular α-subunit and a single-pass transmembrane β-subunit. The extracellular portion of c-Met comprises a large *N*-terminal semaphoring (SEMA) domain composed of the full-length α-subunit connected by a disulfide bridge to the *N*-terminal portion of β-subunit, a small plexin, semaphoring and integrin (PSI) domain and four immunoglobulin-like regions in plexins and transcription factors (IPT) domains. The intracellular moiety of c-Met comprises three segments: a juxtamembrane segment (JM) (Nakayama et al., 2013), a tyrosine kinase (TK) domain and a *C*-terminal docking (Figure 1A; Jeon and Lee, 2017; Demkova and Kucerova, 2018).

Biological activities of HGF mainly depend on its binding to c-Met (Hartmann et al., 1998). The α-chain of HGF contains a high affinity binding site formed by the *N*-terminal hairpin loop and the first K1 domain that engages with the IPT3 and IPT4 domains of c-Met, and a low-affinity site in the SPH domain of the HGF β-chain that interacts with the SEMA domain of c-Met (Stamos et al., 2004; Holmes et al., 2007). However, another group noted that both NK1/α-chain of HGF engages with the Sema domain (Gherardi et al., 2006). High-affinity binding of HGF to c-Met leads to recruitment of key adaptor proteins and intracellular molecules (e.g., GAB1, STAT3, PI3K) and subsequent activation of several downstream signaling pathways to promote cell proliferation, invasion, survival and motility, which have been comprehensively studied (Boccaccio et al., 1998; Maroun et al., 1999; Fan et al., 2005).

Of interest is that the high affinity binding site of HGF for c-Met, as mentioned above, which is also capable of recognizing heparin, an unbranched, heavily sulfated polysaccharides composed of a repeated disaccharide motif. HGF mutants generated by deletion of the *N*-terminal hairpin loop or the K2 domain show reduced apparent affinities for heparin (Hartmann et al., 1998); whereas deletion of K1, K3 or K4 shows little or no effect upon heparin binding. Thus, the heparin-binding site of HGF appears to be formed, partly if not all, by the *N*-terminal hairpin loop and the K2 domain. As those same domains are critical for c-Met binding and HGF/c-Met signaling, it has been implicated that heparin and its analogs heparan sulfate may act as co-receptors of HGF by stabilizing the HGF-c-Met complex, which makes the engagement between HGF and c-Met kinetically more favorable (Holmes et al., 2007).

Both pro-HGF and pro-c-Met undergo post-translational modifications, including glycosylation, disulfide bonds formation and proteolytic cleavage, to generate their mature forms. The most common type of post-translational modifications for RTKs is *N*-glycosylation via transferring an oligosaccharide chain to an asparagine residue of the protein (Giordano et al., 1989; Contessa et al., 2008). C-Met is believed to have 13 putative *N*-glycosylation sites (Asn45, Asn106, Asn149, Asn202, Asn399, Asn405, Asn607, Asn635, Asn785, Asn879, Asn930, Asn998 and Asn1171) within its extracellular domain (Cao et al., 2009; Chen et al., 2013; Bollineni et al., 2018). In addition, experimental evidence implied that there is one potential *O*-glycosylation site in the extracellular domain of c-Met (Wu et al., 2013; Table 1). Active HGF is also decorated with glycans (Shimizu et al., 1992; Fukuta et al., 2005). It has been shown that HGF may possess 5 potential glycosylation sites (Asn294, Asn402, Thr476, Asn566, and Asn653) (Hara et al., 1993), with one of them being *O*-glycosylated in the α-chain of HGF (Thr476) (Shimizu et al., 1992; Table 2). According to currently available structural data, the crystallographic structure of the HGF β-chain complexed with the c-Met SEMA domain revealed that their glycosylation sites are generally distal in space to the interacting interface of the HGF/c-Met complex, suggesting that glycosylation is not required for the direct interaction between HGF and c-Met (Figure 1B). However, detailed information regarding the specific glycosylation sites and glycan composition of c-Met and HGF as well as their associated biological roles still remains largely unknown.

**TABLE 1:** The glycosylation profile of c-Met.

Glycosylation site	Glycosylation mode	Glycan composition^#^
Asn45	*N*-linked	Hex_(n__=__4–7)_
Asn106	*N*-linked	HexNAc_(n__=__3–6)_
Asn149	*N*-linked	NeuAc_(n__=__1–3)_**_;_**
Asn202	*N*-linked	Hex_(n__=__5–6)_
Asn399	*N*-linked	HexNAc_(n__=__3–6)_
Asn405	*N*-linked	dHex_(n__=__1–2)_
Asn607	*N*-linked	NeuAc_(n__=__0–2)_
Asn635	*N*-linked	
Asn785	*N*-linked	
Asn879	*N*-linked	
Asn930	*N*-linked	
Asn998	*N*-linked	
Asn1171	*N*-linked	
N/D	*O*-linked	N/D

*^#^Hex, hexose; dHex, deoxyhexose; HexNAc, *N*-Acetylhexosamine; NeuAc, *N*-Acetyl-Neuraminic Acid; N/D, not defined.*

**TABLE 2:** The glycosylation profile of HGF.

Glycosylation site	Glycosylation mode	Glycan composition^#^
Asn294	*N*-linked	NeuAc(α2-3)Gal(β1-4)GlcNAc
Asn402	*N*-linked	(β1-2)Man(α1-3)[NeuAc(α2-3)
Asn566	*N*-linked	Gal(β1-4)GlcNAc(β1-2)Man(α1
Asn653	*N*-linked	-6)]Man(β1-
Thr476	*O*-linked	4)GlcNAc(β1-4)GlcNAc
		NeuAc(α2-3)Gal(β1-4)[NeuAc(α2-3)]GalNAc

*^#^Gal, Galactose; Man, Mannose; NeuAc, *N*-Acetyl-Neuraminic Acid; GlcNAc, *N*-Acetylglucosamine; GalNAc, *N*-Acetylgalactosamine.*

## A Brief Overview of Glycans and Protein Glycosylation

All vertebrate cells are coated with glycans, which are in the hub of a great variety of biological activities, including cell-cell crosstalk, signal transduction and host-microbe interactions (Varki and Lowe, 2009; Freeze, 2013; Pinho et al., 2013; Varki, 2017). Glycans comprise of various numbers of monosaccharides connected by glycosidic linkages in either linear or branched form (Ohtsubo and Marth, 2006; Moremen et al., 2012), which is structurally diverse and unique compared to other fundamental cellular macromolecules (proteins, nucleic acids and lipids). The presence of substantial regiochemistries and stereochemistries, together with variations in monosaccharide composition (Yarema and Bertozzi, 2001), contribute to the tremendous diversity of glycans (Springer and Gagneux, 2013). The non-template-driven synthesis of glycans is orchestrated through sequential enzymatic actions of an estimated involvement of 700 glycosidases and glycosyl-transferases in the endoplasmic reticulum (ER) and Golgi, leading to approximately ∼7,000 glycoprotein complexes (Cummings, 2009; Moremen et al., 2012). It has been well documented that glycosylation serves as a critical determinant facilitating the functional diversity of proteins (Ohtsubo and Marth, 2006; Marth and Grewal, 2008; Reis et al., 2010). Overall, more than half of proteins are estimated to be glycosylated in their life cycle, compared to that only 30% of proteins are phosphorylated (Zielinska et al., 2012). Noteworthy, The glycosylation content of a given protein is determined by the presence and frequency of glycosylation sites in its own amino acid sequence, as well as the expression and activities of specific glycosyl-regulatory enzymes within the biological context (Marth and Grewal, 2008).

The abundant and commonly occurring modes of eukaryotic protein glycosylation arise from *O*- and *N*-linkages. In *N*-linked glycosylation, a 14-monosaccharide glycan block (Glc_3_Man_9_GlcNAc_2_) is covalently linked to the asparagine residue of the aglycone part of the protein, usually involving a consensus peptide sequence Asn-X-Ser/Thr (where X stands for any amino acid except proline) (Stanley et al., 2015). The newly synthesized glycoproteins then undergo the folding procedure followed by further modifications of glycans in ER and Golgi, eventually giving rise to three main types of *N*-linked glycans: the high mannose, the hybrid, and the complex types (Moremen et al., 2012; Stanley et al., 2015). In *O*-glycosylation, either a GalNAc moiety (commonly referred to as mucin-type) or a GlcNAc moiety is added to the hydroxyl group of a serine or threonine residue in the backbone of proteins (Jensen et al., 2010; Slawson and Hart, 2011; Li et al., 2020). In contrast to *N*-glycosylation, the synthesis of *O*-glycans is undertaking through a stepwise mode by adding a single monosaccharide moiety (Guzman-Aranguez and Argueso, 2010; Brockhausen and Stanley, 2015). The *N*- and *O*-glycans are *in vivo* further elongated to heterogeneous carbohydrate chains by the addition of various monosaccharides terminated in usually mannose, fucose and sialic acid, along with additional diversification in chemical substitutions, such as sulfation and acetylation.

Abnormal protein glycosylation are commonly found in cancers, which can be utilized as a hallmark of cancer progression and metastasis (Hakomori and Cummings, 2012). Some of the most widely-utilized serological biomarkers for cancer diagnosis as well as cancer progression are glycoproteins, such as PSA in patients with prostate cancer and CA19-9 in patients with pancreatic cancer (Pinho and Reis, 2015). The most common types of altered glycosylations in cancers are known as sialylation, fucosylation, *O*-glycan truncation, and *N*- and *O*-linked glycan branching (Marth and Grewal, 2008; Pinho and Reis, 2015). These alterations are generally associated with abnormal gene expression of a multitude of enzymes involved in the glycan synthesis as well as post-synthetic modifications. For example, expression of glycans is tightly correlated with proper expression and localization of the relevant glycosyltransferases and glycosidases in the Golgi apparatus (Potapenko et al., 2015; Vojta et al., 2016). Further, glycan expression can be influenced by the variability of acceptors together with the availability and abundance of saccharide donors and cofactors (Reis et al., 2010).

## Heparan Sulfate Proteoglycans

As a key component of ECM, HSPGs comprise of a transmembrane or secreted protein core to which one or more heparan sulfate (HS) chains are covalently attached. HSPGs are one of the most highly negatively charged biopolymers occurred naturally, collaborating with other ECM components to facilitate the ECM remodeling and structural integrity (Iozzo, 2005). The varied number of attached HS chains, together with the sulfation composition along the HS chains, maximizes the structural heterogeneity of HSPGs. Of relevance is that the structural diversity of HS modulates its capability of accommodating a variety of binding partners, such as growth factors and chemokines, which is central to the diverse biological roles of HSPGs, leading to activation of downstream signal cascades and promotion of cell proliferation, tumor cell dissemination, inflammation and angiogenesis (Iozzo and San Antonio, 2001; Goodall et al., 2014). Heparanase (HPSE) is defined as the only known endo-*β*-D-glucuronidase that catalyzes HS hydrolysis to date (Peterson and Liu, 2010, 2013). Previous studies have demonstrated that HPSE is able to act in either a consecutive or a gapped cleavage mode depending on the saccharide sequences of HS, which enables the efficient release of distinct bioactive molecules, such as HGF, from HSPGs (Peterson and Liu, 2013).

## Gangliosides

Gangliosides are a class of glycosphingolipids widely expressed on the cell surface in mammal species, with a functional glycan head group typically containing sialic acids and a ceramide tail that is anchored in the membrane (Oblinger et al., 2003). Gangliosides are involved in many biological events, mainly including cellular recognition, cell-cell communication and signal transduction (Yu et al., 2011). As functions of gangliosides are practically determined by their glycans, changes in expression levels and profiles of gangliosides can be largely attributed to the dynamic regulation of ganglioside synthases, a group of glycosyltransferases (Ishii et al., 2007). RTKs, including c-Met, are commonly found to colocalize with gangliosides with in glycolipid-enriched microdomains. As a result, c-Met activity can be regulated by gangliosides, which act by different mechanisms (Ferreira et al., 2018). Significantly, the regulation of c-Met by gangliosides seems to be determined by the glycan profile of gangliosides (Cazet et al., 2012). In bladder epithelial cells, ganglioside GM_3_ and GM_2_ downregulate the trans-phosphorylation of c-Met, which in turn impairs the recruitment of Grb2 as well as activation of downstream pathways (Todeschini et al., 2008). Ganglioside GD_1a_ was also demonstrated to inhibit HGF-induced motility through the suppression of phosphorylation of c-Met in mouse osteosarcoma cells (Hyuga et al., 2001). On the other hand, activation of c-Met was reported to be modulated by ganglioside GD2 and GD3 in breast cancer cells, leading to enhanced cell migration and proliferation (Cazet et al., 2012; Table 3).

**TABLE 3:** Glycoconjugates involved in regulation of the HFG/c-Met axis.

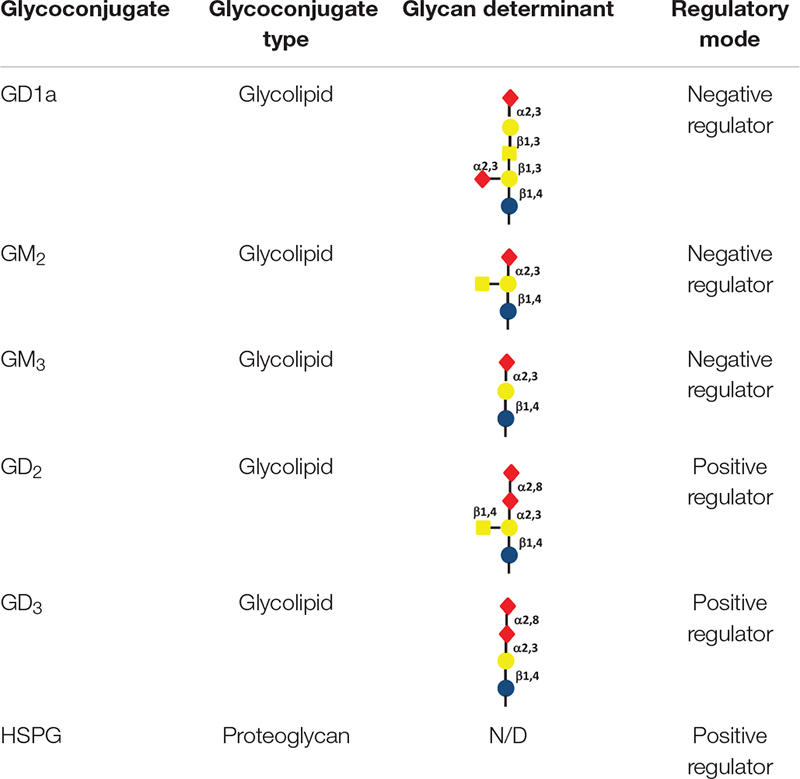

*N/D, not defined; 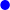 Glucose; 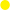 Galactose; 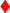 Neu5Ac; 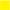
*N*-Acetylgalactosamine.*

## Aberrant HGF-c-Met Signaling

Dysregulated HGF-c-Met activation is observed in a wide range of malignant tumors (Di Renzo et al., 1995b; Fukuura et al., 1998; Lengyel et al., 2005; Porta et al., 2013; Cao et al., 2015; Lam et al., 2016). For instance, increased mRNA level of HGF has been reported in approximately ∼70% tumors (Di Renzo et al., 1995a; Maroun et al., 1999). In malignances, HGF is commonly synthesized and released by surrounded stromal cells, including cancer-associated fibroblasts (CAFs) and tumor-associated microphages (TAMs) (Matsumoto and Nakamura, 2006; Lorusso and Ruegg, 2008), triggering c-Met activation in a paracrine manner. Further, autocrine production of pro-HGF by tumor cells is also observed in multiple tumors (Fukuura et al., 1998; Seidel et al., 1998; Porta et al., 2013; Liu, 2015) and HGF is essential for the crosstalk between tumor cells and stromal cells (Gherardi et al., 2006; Luraghi et al., 2014; Pallangyo et al., 2015). Fibroblasts-restricted deletion of IKKβ increases HGF production, which leads to enhanced intestinal tumorigenesis *in vivo*. Higher secretion level of HGF is observed in the culture medium of CAFs isolated from hepatocellular carcinoma (H-CAFs) than that in the culture medium of CAFs from normal skin fibroblasts. Blockade of HGF highly reduces H-CAFs proliferation, indicating that HGF is a promising target of antitumor therapies. Under pathological conditions, overexpression of c-Met at both mRNA and protein levels has also been shown in several solid tumor progression (Di Renzo et al., 1995b; Lengyel et al., 2005; Cao et al., 2015). Of interest is that down-regulated internalization and degradation of c-Met also lead to oncogenic activation (Peschard et al., 2001; Hu et al., 2015). Oncoprotein protein kinase C (PKC) plays an important role in c-Met endosomal process, while targeted disruption of PKCε blocks the c-Met-JNK-paxillin signaling pathway in hepatocellular carcinoma cell (HCC). Oncogene fusion protein TPR-Met inhibited binding of E3 ligase Cbl to the JM domain, which is beneficial for ubiquitin-mediated proteasome degradation of c-Met.

## The Glycosylated Impact Upon the HGF-c-Met Axis

RTKs, including c-Met, have been considered as potential therapeutic targets in numerous cancers, such as HCC (Bouattour et al., 2018), colon cancer (Qian et al., 2009), head and neck cancer (Arnold et al., 2017), lung cancer (Miranda et al., 2018) and glioblastoma (Cruickshanks et al., 2017). It has been proposed that disruption of *N*-linked glycosylation could lead to a decreased expression level of mature RTKs and thus interfere with the RTK signaling cascades in cancers. Previous evidence suggested that tunicamycin (an inhibitor of *N*-glycosylation) treatment impedes proper cleavage and phosphorylation of c-Met in GTL-16 cells (Giordano et al., 1989), and leads to the cytoplasmic retention of c-Met in HCC cells. Disruption of c-Met glycosylation promotes proteasome degradation (Chen et al., 2013) and attenuates downstream signaling cascades including ERK, AKT and STAT3, therefore affecting cell proliferation, migration, metabolism or angiogenesis (Qian et al., 2009; Wu et al., 2013). α2,6-hyposialylation inhibition of c-Met that is mediated via ST6Gal-I-knockdown leads to impaired cell motility in colon cancer cell line HCT116 (Qian et al., 2009). In addition, upregulation of α1,6-fucosyltransferase (Fut8) is involved in c-Met regulation in HCC (Wang et al., 2015). Knockout of Fut8 leads to AKT and ERK signaling attenuation upon HGF stimulation and cell proliferation *in vitro*. Limited xenograft tumor growth *in vivo* with Fut8 KO HepG2 is also observed. To further explore the role of Fut8 in carcinogenesis, Fut8-/- mice with DEN/PB treatment are used to induce HCC. Compared to Fut8+/+ mice, Fut8-/- mice show limited liver carcinogenesis and liver regeneration (Wang et al., 2015), indicating the essential role of glycosylation on HGF-c-Met signaling and tumor progression.

Early studies indicated that *O*-linked glycosylation is another potential post-translational modification of c-Met. Wu et al. (2013) found c-Met has one potential *O*-glycosylation site within the extracellular domain. Furthermore, C1GALT1, a critical mucin-type *O*-glycosyltransferase localized in the Golgi apparatus (Tu and Banfield, 2010; Petrosyan et al., 2012), is responsible for *O*-glycan modification of c-Met by enhancing c-Met dimerization and activation following HGF stimulation *in vivo* and *in vitro* (Wu et al., 2013). siRNA-mediated deletion of C1GALT1 inhibits c-Met phosphorylation and dimerization, resulting in attenuated abilities of regulating HCC cell proliferation, migration, invasion and growth in SCID mouse model (Nakamura et al., 1984). However, it seems that secreted HGFs with or without glycosylation do not display different physicochemical characteristics and biological activity. Fukuta et al. purified glycosylation-deficient HGFs in the form of non-glycosylated α-chain, β-chain, or α-β-chain, respectively. According to their findings, non-glycosylated HGF had the same potency as secreted glycosylated HGF upon inducing c-Met tyrosine phosphorylation and downstream signaling activation, although glycosylation-deficiency inhibited post-transcriptional biosynthesis of HGF (Fukuta et al., 2005).

**FIGURE 2:**
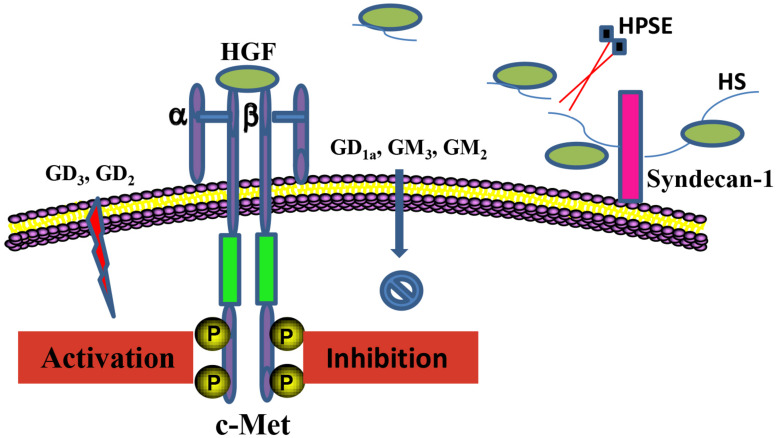
Regulation of the HGF/c-Met axis by glycoconjugates. HSPGs and gangliosides in the local cellular environment are able to regulate HGF/c-Met activity. Anchored HGF are released from HS chains of HSPGs upon the cleavage by HPSE, which mediates the interaction between HGF and c-Met. Regulation of the HGF/c-Met by gangliosides is critically determined by the composition of the ganglioside glycan chains.

Glycoconjugates including HSPGs and gangliosides in the local cellular environment also play important roles in regulation of HGF/c-Met activity (Figure 2). HSPGs and its sole enzyme HPSE can directly modulate HGF function. HGF expression by myeloma cells is significantly elevated by HPSE, which facilitates the HGF binding by myeloma cell surface syndecan-1, a member of HSPGs, leading to HGF-enhanced myeloma tumor cell growth (Deakin and Lyon, 1999). HS chains of syndecan-1 is also capable of trapping HGF at the cell surface, and thereby promotes the engagement between HGF and c-met (Derksen et al., 2002). Further, c-Met signaling in osteoblasts can be stimulated by shedding syndecan-1/HGF complexes (Seidel et al., 2000). In addition, it has been reported that NK4 of HGF binds to perlecan, another member of HSPGs, leading to impairment of fibronectin assembly in a c-Met-independent manner, therefore inhibiting anchorage-dependent signaling (Sakai et al., 2009). Regulation of HGF/c-Met by gangliosides is principally determined by their glycan composition. Overall, monosialogangliosides that only contain one sialic acid are suggested to negatively regulate the HGF-c-Met signaling pathway, whereas disialogangliosides that contain two sialic acid moieties, except for GD_1a_, mediate the activation of c-Met (Oblinger et al., 2003; Cazet et al., 2012; Ferreira et al., 2018). Nevertheless, the molecular mechanisms by which gangliosides regulate the HGF-c-Met are yet to be elucidated.

## Conclusion and Future Perspectives

Upon HGF-induced activation, aberrant c-Met activation facilitates enhanced tumor cell growth, angiogenesis and invasion in cancer, which is overall associated with poorer survival, suggesting that the c-Met/HGF axis is a promising therapeutic target against malignancies. Extracelluar domains of both c-Met and HGF are heavily glycosylated, with the primary glycosylation mode being *N*-linked. Disruption of *N*-linked glycosylation appears to downregulate the expression level of several RTKs, including c-Met, and subsequently suppress the downstream signaling pathways. However, though multidisciplinary approaches have been applied to evaluate and develop effective therapeutics targeting the c-Met/HGF axis, the glycosylation machinery regulating the c-Met/HGF axis is less explored.

Due to the fact that glycosylation also plays key physiological roles under normal circumstances, a question may arise that is it a practical target in cancer therapy? According to currently available data and also due to the non-template-driven nature of glycans, it is likely that glycosylation-related inhibitors targeting the c-Met/HGF axis, irrespective of their nature and source, i.e., independently from being glycolsyltransferase inhibitors or nucleoside antibiotics, will be likely used in combination with other therapeutics in the treatment.

Another major challenge in the glycobiology field is to decipher the structural complexity of glycans. Glycomics, compared to genomics and proteomics, has lagged far behind, partly due to analytical difficulties arising from the diverse and heterogenous profile of glycans. Recent advance in analytical techniques has led to detailed characterizations of the glycoproteome of cancer cells. Given the specific glycan information of HGF and c-Met still remains elusive, the acquisition of those relevant knowledge would provide potent opportunities for scouting novel biomarkers and drugs targeting the c-Met/HGF axis in the foreseeable future.

## Author Contributions

HL, LW, and XY proposed the study. XH and FT performed the research and wrote the first draft. All authors contributed to the interpretation of the study and to further drafts.

## Conflict of Interest

The authors declare that the research was conducted in the absence of any commercial or financial relationships that could be construed as a potential conflict of interest.
